# Segment-Specific Gut Microbiome and Bile Acid Profiles in Grazing and Stall-Fed Yaks

**DOI:** 10.3390/microorganisms14091960

**Published:** 2026-09-04

**Authors:** Qiuyue Li, Jiaming Wang, Zhilong Wang, Chenxu Cheng, Shuting Bao, Shatuo Chai, Dongwen Dai, Xun Wang, Qizhu Song, Yongwei Chen, Jiaying Lv, Yijuan Ma, Tashi Sonam, Junchao Qiu, Shuxiang Wang

**Affiliations:** 1 College of Animal Husbandry and Veterinary Sciences, Qinghai University, Xining 810016, China; 17713822124@163.com (Q.L.);; 2 Qinghai Provincial Yak Engineering and Technology Research Center, Xining 810016, China; 3 Qinghai Provincial Animal Husbandry Station, Xining 810016, China; 4 Tibet Pure Land Dairy Co., Ltd., Lhasa 850000, China

**Keywords:** yak, feeding system, gut microbiota, bile acid, metagenomics

## Abstract

The yak is an iconic ruminant of the Qinghai-Tibet Plateau, yet segment-specific variation in its intestinal microbial functional potential and bile acid profiles under different feeding systems remains insufficiently characterized. Six healthy adult male yaks with similar body weights (320 ± 30 kg) were assigned to grazing (G) or stall-feeding (S) systems, with three animals per group, for a 90-day trial comprising a 10-day adaptation period and an 80-day formal experimental period. The individual yak was considered the experimental unit, and intestinal segments sampled from the same animal were treated as repeated observations. Liver tissue and digesta from the duodenum, ileum, cecum, and colon were analyzed using targeted bile acid metabolomics and shotgun metagenomics. Principal coordinate analysis based on Bray-Curtis dissimilarities showed segment-associated clustering of microbial communities, with PCo1 and PCo2 explaining 65.5% and 18.9% of the total variation, respectively. ANOSIM identified a significant intestinal-segment effect on microbial community composition (*R* = 0.2208, BH-FDR = 0.0144), whereas the overall feeding-system effect was not significant (*R* = 0.3747, BH-FDR = 0.1200). No statistically significant feeding-system differences were detected in Shannon, Simpson, Chao1, or ACE indices within individual intestinal segments (BH-FDR ≥ 0.800), and PERMDISP detected no significant differences in within-group dispersion (BH-FDR ≥ 0.1682). Bacillota and Bacteroidota were the dominant phyla. Descriptive functional profiling showed higher mean ileal abundances of GH2 (0.0035 vs. 0.0026), GH3 (0.0029 vs. 0.0024), and GH43 (0.0021 vs. 0.0013) in grazing yaks, whereas the starch-associated GH13 family showed its highest mean abundance in the colon of stall-fed yaks. These metagenomic patterns represent predicted genomic functional potential rather than gene expression, enzyme activity, or metabolic flux. Cecal total bile acid concentration showed a nominal between-group difference (unadjusted Welch’s *p* = 0.0109), but this difference did not remain significant after correction across the five anatomical sites (BH-FDR = 0.0545). In the colon, stall-fed yaks had a lower conjugated-to-unconjugated bile acid ratio and a higher secondary-to-primary bile acid ratio than grazing yaks (BH-FDR < 0.05). Feeding-system-associated descriptive patterns were observed in predicted microbial functional profiles, whereas statistically supported between-group differences were limited mainly to selected colonic bile acid ratios. Given the limited animal-level replication, these findings should be considered exploratory.

## 1. Introduction

The yak (*Bos grunniens*) is a species unique to the Qinghai–Tibet Plateau. It serves not only as an indispensable production resource for local herders but also as a crucial component in maintaining the ecological balance of alpine grasslands [1]. Traditionally, the grazing system has served as the dominant feeding strategy to which yaks have long adapted, enabling them to efficiently utilize the natural resources of highland pastures [2]. However, with increasing market demand and the implementation of ecological protection policies, intensive stall-feeding systems are gradually being promoted to enhance production efficiency and achieve a sustainable balance between livestock and grassland resources [3]. While this transformation improves productivity, it substantially alters the dietary structure-shifting from diverse natural forages to concentrated feeds-which may, in turn, profoundly influence the digestive metabolism and overall health of yaks [4].

Bile acids (BAs) play a crucial role in the digestive and metabolic processes of yaks. As the end products of hepatic cholesterol metabolism, their classical function is to emulsify lipids and facilitate the absorption of fats and fat-soluble vitamins. In recent years, bile acids have been recognized as important signaling molecules. By activating pathways such as the farnesoid X receptor (FXR) and the G protein-coupled receptor TGR5, bile acids broadly regulate host glucose homeostasis, lipid metabolism, energy balance, and immune-inflammatory responses [5]. The dynamic equilibrium of the bile acid pool-particularly the ratio between primary bile acids (such as cholic acid, CA, and chenodeoxycholic acid, CDCA) and their gut microbiota-derived secondary bile acids (such as deoxycholic acid, DCA, and lithocholic acid, LCA) is vital for maintaining metabolic homeostasis. During this process, bacterial bile salt hydrolases (BSHs) secreted by the intestinal microbiota deconjugate conjugated bile acids, which are subsequently transformed from primary to secondary forms via microbial 7α-dehydroxylase activity [6]. Microbial 7α-dehydroxylation is restricted to a subset of gut microorganisms harboring the relevant bile-acid-inducible (*bai*) machinery, and the detection of individual bile-acid-related genes does not by itself demonstrate active pathway-level conversion. These microbially driven transformations not only alter the physicochemical properties of bile acids but also affect their signaling activity. Therefore, intestinal bile acid profiles may reflect the combined influences of microbial transformation and host physiological processes, including hepatic synthesis, intestinal absorption, and enterohepatic circulation. Feeding system is an important environmental factor associated with variation in intestinal microbial communities. In ruminants, the transition from diverse, fiber-rich natural forage to concentrate-based diets may alter substrate availability, intestinal physicochemical conditions, and microbial functional potential [7].

Previous studies have mainly examined the effects of feeding systems on yak growth performance, meat quality, and gut microbial composition [8]. However, within-animal, segment-resolved studies integrating shotgun metagenomic functional profiles with targeted bile acid measurements across multiple intestinal regions of the yak remain limited. The novelty of the present study lies in integrating these two data layers across the duodenum, ileum, cecum, and colon within the same animals, thereby providing a spatially resolved view of microbiome-bile acid variation rather than establishing causal effects of feeding system on microbial or bile acid metabolism. In particular, it is still unclear whether intestinal segment represents the dominant source of variation in the yak gut microbiome and bile acid landscape, and whether grazing and stall-feeding are associated with selective differences within individual intestinal segments. In this exploratory study, we integrated shotgun metagenomic sequencing and targeted bile acid metabolomics to characterize the duodenum, ileum, cecum, and colon of grazing and stall-fed yaks. The objectives were to: (1) characterize spatial variation in microbial community composition, predicted carbohydrate-degrading functions, and bile acid profiles along the intestinal tract; (2) compare grazing and stall-fed yaks within each intestinal segment; and (3) identify exploratory associations among dominant microbial genera, predicted bile-acid-related genes, and bile acid composition. Rather than establishing causal regulatory mechanisms, this study was designed to generate testable hypotheses regarding segment-specific microbiome–bile acid relationships in the yak intestine.

## 2. Materials and Methods

### 2.1. Experimental Design and Feeding Management

All experimental protocols and procedures were reviewed and approved by the Animal Experiment Committee of Qinghai University (approval No. QHU20240915). All animal experiments were conducted in accordance with the Experimental Animal Care and Use Guidelines of China. The experiment was conducted at the breeding base and pasture of Qinghai Zhonghui Agriculture and Animal Husbandry Technology Co., Ltd., located in the National Agricultural Industrial Park of Zeku County, Huangnan Tibetan Autonomous Prefecture, Qinghai Province, China. Six healthy adult male yaks with similar body weights (320 ± 30 kg) were assigned to either the grazing (G) or stall-feeding (S) group, with three animals per group. The individual yak was considered the experimental unit. No formal a priori power calculation was performed because the study was designed as an exploratory investigation. Given the limited animal-level replication (n = 3 per feeding system), between-group comparisons were interpreted cautiously and were considered hypothesis-generating. Four intestinal segments were sampled from each animal; therefore, segment-level samples from the same yak were treated as repeated observations rather than independent biological replicates. The grazing group was allowed free access to natural alpine meadow pasture daily from 09:00 to 17:00 and had free access to water. No quantitative data on pasture botanical composition, nutrient composition, or individual forage intake were available. The stall-fed group was housed indoors and was fed a concentrate mixture together with oat hay. On a dry-matter basis, the oat hay contained 3.29% crude protein, 2.19% ether extract, 49.8% neutral detergent fiber, 31.3% acid detergent fiber, 0.50% calcium, and 0.08% phosphorus. The ingredient composition and nutrient levels of the concentrate mixture are shown in Table 1. The entire trial lasted for 90 days, consisting of a 10-day pretrial adaptation period followed by an 80-day formal experimental period.

### 2.2. Sample Collection

At the end of the experiment, all yaks were slaughtered according to the approved experimental protocol. Liver tissue samples and digesta from the duodenum, ileum, cecum, and colon were collected separately. Different intestinal segments were handled independently using sterile instruments to minimize cross-contamination. The collected digesta samples were transferred into sterile cryogenic tubes and immediately frozen in liquid nitrogen. All samples were subsequently stored at −80 °C until further analysis.

### 2.3. Targeted Bile Acid Metabolomics Analysis

#### 2.3.1. Bile Acid Extraction

Targeted bile acid metabolomics analysis was performed by Metabo-Profile Biotechnology (Shanghai) Co., Ltd. (Shanghai, China). Bile acid standards were obtained from Steraloids Inc. (Newport, RI, USA) and TRC Chemicals (Toronto, ON, Canada), with additional standards custom-synthesized in the laboratory. Isotope-labeled bile acid internal standards were obtained from C/D/N Isotopes Inc. (Pointe-Claire, QC, Canada) and Steraloids Inc. (Newport, RI, USA). Analytical-grade ammonium acetate was purchased from Sigma-Aldrich (St. Louis, MO, USA), whereas chromatographic-grade methanol, isopropanol, acetonitrile, and glacial acetic acid were purchased from Thermo Fisher Scientific (Fair Lawn, NJ, USA). Quality control (QC) samples were prepared by pooling aliquots from all samples and were analyzed together with experimental samples to monitor analytical stability and reproducibility. QC performance was evaluated using QC correlation analysis and multivariate quality-control assessments provided by the metabolomics platform. Approximately 10 mg of liver tissue or 5 mg of freeze-dried intestinal content was accurately weighed and homogenized in an acetonitrile/methanol (8:2, *v*/*v*) mixed solvent containing isotope-labeled bile acid internal standards for quality control and compensation of matrix effects. The homogenate was then centrifuged using a Microfuge 20R centrifuge (Beckman Coulter, Inc., Indianapolis, IN, USA) at 13,500× *g* for 20 min at 4 °C, and the supernatant was collected. For liver samples, the obtained supernatant was freeze-dried using a lyophilizer (Labconco, Kansas City, MO, USA). The dried residue was reconstituted in a 1:1 (*v*/*v*) mixture of acetonitrile/methanol (8:2, *v*/*v*) and ultrapure water, followed by another centrifugation at 13,500× *g* for 20 min at 4 °C. The injection volume for analysis was 5 µL. Quantification was performed using calibration curves generated from bile acid standards at multiple concentration levels, and bile acid concentrations were calculated based on the corresponding regression equations.

#### 2.3.2. Chromatographic Conditions

Chromatographic separation was performed using an ACQUITY UPLC CORTECS C18 VanGuard pre-column (2.1 × 5 mm) and analytical column (2.1 × 100 mm, 1.6 µm; Waters Corporation, Milford, MA, USA). The column temperature was maintained at 30 °C, and the autosampler temperature was maintained at 10 °C. The mobile phase consisted of solvent A (10 mM ammonium acetate containing 0.25% acetic acid) and solvent B (acetonitrile:methanol:isopropanol, 8:1:1, *v*/*v*/*v*). Gradient elution was performed as follows: 0–0.3 min, 5% B; 0.3–0.5 min, 5–10% B; 0.5–2 min, 10–15% B; 2–3 min, 15–30% B; 3–6 min, 30% B; 6–8 min, 30–35% B; 8–9 min, 35–40% B; 9–10 min, 40% B; 10–15 min, 40–75% B; 15–15.5 min, 75–100% B; 15.5–16.2 min, 100% B; 16.2–16.3 min, 100–5% B; and 16.3–17 min, 5% B. The flow rate was 0.40 mL/min, and the injection volume was 5 µL.

#### 2.3.3. Mass Spectrometry Conditions

An electrospray ionization (ESI) source was operated in negative ion mode. The ionization voltage was set at 4.5 kV, with the source temperature maintained at 500 °C. The other parameters were as follows: curtain gas pressure, 30 psi; collision gas pressure, medium; nebulizer gas pressure, 50 psi; auxiliary heating gas pressure, 50 psi.

### 2.4. Metagenomic Sequencing and Bioinformatics Analysis

Genomic DNA libraries were constructed following standard DNBSEQ library preparation procedures. DNA was fragmented to approximately 350 bp, followed by end repair, adapter ligation, PCR amplification, and quality assessment. Qualified libraries were circularized and amplified by rolling circle amplification to generate DNA nanoballs, which were sequenced on the DNBSEQ-T7 platform (MGI Tech Co., Ltd., Shenzhen, China) by Novogene Bioinformatics Technology Co., Ltd. (Beijing, China). Paired-end sequencing was performed with a read length of 150 bp (PE150).

Raw paired-end reads were quality-filtered using fastp (v0.23.1), and host-derived reads were removed using Bowtie2 (v2.5.4) against the yak reference genome. Sequencing generated an average of approximately 25.1 million clean read pairs (7.53 Gb) per sample, with a mean Q30 value of 98.43%. After host-read removal, an average of 5.68 Gb of non-host data per sample was retained. Non-host reads were assembled using MEGAHIT (v1.2.9), and scaftigs shorter than 500 bp were excluded. Across the 24 samples, the assembled metagenomes showed an average total length of 520.53 Mb, 508,950 scaftigs, and an N50 value of 1020 bp per sample [9]. Open reading frames were predicted from scaftigs ≥500 bp using GeneMark.hmm (v2.1), and predicted ORFs shorter than 100 nt were discarded. CD-HIT (v4.5.8) with a 95% sequence identity threshold and 90% alignment coverage was used to construct a nonredundant gene catalogue containing 8,895,706 genes [10]. Clean reads from each sample were mapped to the nonredundant gene catalogue using Bowtie2 (v2.5.4), and gene abundance was calculated based on mapped-read counts and gene length. Core–pan gene rarefaction curves were generated by randomly subsampling increasing numbers of samples and calculating the numbers of nonredundant genes observed across the resulting sample combinations. Taxonomic annotation was performed by aligning the nonredundant gene catalogue against the Micro_NR database (version 2024.03) using DIAMOND (v2.1.9). Functional annotation was performed against the KEGG (version 2024.03), eggNOG (version 5.0), and CAZy (version 2024.03) databases using DIAMOND (v2.1.9). Taxonomic and predicted functional abundance profiles were generated by combining the annotation results with gene abundance data. Metagenomic functional profiles, including CAZy and bile-acid-related KEGG annotations, were interpreted descriptively as indicators of predicted genomic functional potential rather than as evidence of gene expression, enzyme activity, or metabolic flux.

### 2.5. Data Processing and Statistical Analysis

Peak detection, integration, and quantification were performed using MassLynx software (v4.1, Waters, Milford, MA, USA), whereas the analytical platform was an AB Sciex QTRAP 6500+ UPLC-MS/MS system(AB Sciex LLC, Framingham, MA, USA). Multivariate analyses were conducted using the iMAP platform (v1.0, Metabo-Profile, Shanghai, China). The individual yak was considered the experimental unit for all statistical analyses. Because the duodenum, ileum, cecum, and colon were sampled from the same animal, segment-level observations from the same yak were treated as repeated observations rather than independent biological replicates. Comparisons between grazing and stall-fed yaks within each anatomical site were therefore based on three animals per feeding system. For analyses involving multiple intestinal segments, the repeated-measures structure was accounted for through restricted permutation procedures where applicable. Because of the limited number of independent animals (n = 6), mixed-effects models were not applied for confirmatory inference, as reliable estimation of random effects and interaction terms would be constrained. Total bile acid concentrations were compared between feeding systems separately within each anatomical site using Welch’s *t*-tests. Given the limited sample size (n = 3 per feeding system), normality assessments were not used as a basis for selecting statistical tests. Welch’s *t*-tests were used for exploratory comparisons, and the resulting *p* values were reported as unadjusted values with *p* < 0.05 considered the nominal significance threshold. To account for multiple comparisons across the five anatomical sites, these five *p* values were additionally adjusted using the Benjamini–Hochberg false discovery rate procedure. Conjugated-to-unconjugated and secondary-to-primary bile acid log-ratios were compared using Welch’s *t*-tests, and the resulting 10 *p* values were adjusted using the Benjamini–Hochberg false discovery rate procedure. Significance for the ratio analyses was defined as BH-FDR < 0.05. Within each intestinal segment, Spearman rank correlations were calculated between the 15 most abundant genera and bile acid concentrations transformed as log10(x + 1 × 10^−6^), using data from all six animals. Owing to the small sample size (n = 6) and the large number of genus–metabolite pairs examined, the correlation analysis was considered exploratory and hypothesis-generating. Correlation coefficients were not interpreted as evidence of causality. CAZy family abundances and bile-acid-related KEGG ortholog abundances were summarized descriptively as group means within each intestinal segment. Given the limited animal-level replication (n = 3 per feeding system), no confirmatory differential-abundance testing was performed for these functional profiles. These metagenomic measurements were interpreted as predicted genomic functional potential rather than as evidence of gene expression, enzyme activity, or metabolic flux. Alpha-diversity indices were compared between feeding systems separately within each intestinal segment using two-sided exact permutation tests, followed by Benjamini–Hochberg correction across the 16 resulting comparisons. Bray–Curtis dissimilarities were used for principal coordinate analysis, ANOSIM, and PERMDISP. For ANOSIM, intestinal-segment permutations were restricted within animal identity, whereas feeding-system labels were permuted at the animal level. *p* values from the six ANOSIM tests (one overall intestinal-segment test, one overall feeding-system test, and four segment-specific feeding-system comparisons) were adjusted using the Benjamini–Hochberg procedure. PERMDISP was performed using 9999 permutations, and *p* values from the five dispersion analyses were adjusted using the Benjamini–Hochberg procedure. Unless otherwise stated, data are presented as mean ± SD.

## 3. Results

### 3.1. Gut Microbial Diversity and Community Structure Across Intestinal Segments and Feeding Systems

**Figure 1:**
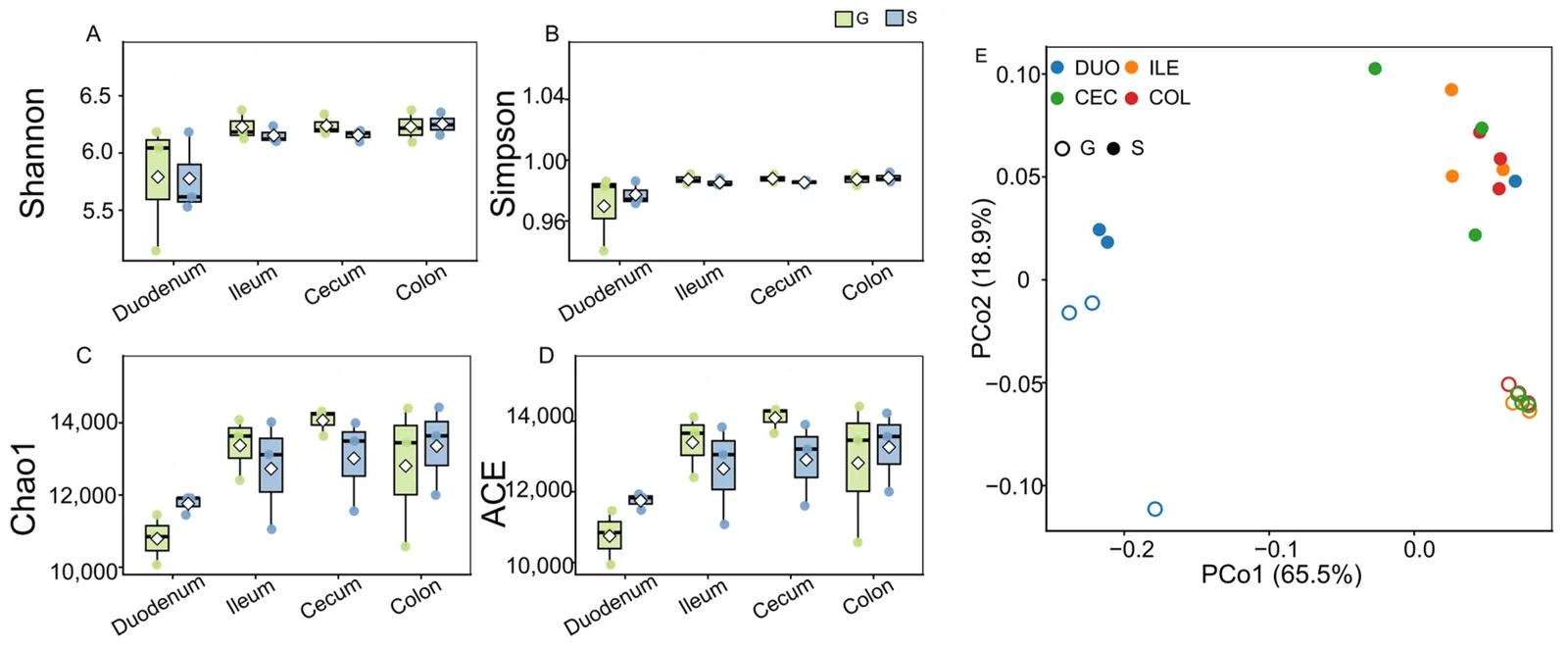
Alpha and beta diversity of gut microbial communities across four intestinal segments in grazing and stall-fed yaks. Digesta samples were collected from the duodenum, ileum, cecum, and colon of grazing yaks (G; n = 3) and stall-fed yaks (S; n = 3). (**A**–**D**) Shannon, Simpson, Chao1, and abundance-based coverage estimator (ACE) indices, respectively. Light-green and light-blue boxes represent G and S, respectively; boxes show the interquartile range, center lines show medians, diamonds show means, and circles represent individual animals. Groups were compared within each intestinal segment using two-sided exact permutation tests. Statistical comparisons were performed using three independent animals per feeding system. *p* values were adjusted across 16 comparisons using the Benjamini–Hochberg procedure, and no comparison was significant (all BH-FDR ≥ 0.800). (**E**) Principal coordinate analysis based on Bray-Curtis dissimilarities. Colors indicate intestinal segments, whereas open and filled circles represent G and S, respectively. PCo1 and PCo2 explain 65.5% and 18.9% of the variation. DUO, duodenum; ILE, ileum; CEC, cecum; COL, colon; PCoA, principal coordinate analysis; BH-FDR, Benjamini–Hochberg false discovery rate.

Alpha-diversity indices, including Shannon, Simpson, Chao1, and ACE, showed no statistically significant feeding-system-associated differences within any intestinal segment (all BH-FDR ≥ 0.800; Figure 1A–D). Principal coordinate analysis based on Bray–Curtis dissimilarities showed segment-associated clustering of microbial communities across the duodenum, ileum, cecum, and colon, with PCo1 and PCo2 explaining 65.5% and 18.9% of the total variation, respectively (Figure 1E). ANOSIM identified a significant intestinal-segment effect on microbial community composition (R = 0.2208, BH-FDR = 0.0144; Table 2). Neither the overall feeding-system effect (R = 0.3747, BH-FDR = 0.1200) nor the segment-specific feeding-system comparisons were statistically significant (all BH-FDR ≥ 0.1200; Table 2). PERMDISP also detected no significant differences in within-group dispersion between feeding systems (all BH-FDR ≥ 0.1682; Table 3). Overall, intestinal segment represented the major source of variation associated with microbial community composition, whereas the feeding system was not significantly associated with alpha diversity, overall community composition, or within-group dispersion.

### 3.2. Taxonomic Composition of Gut Microbiota Across Intestinal Segments and Feeding Systems

**Figure 2:**
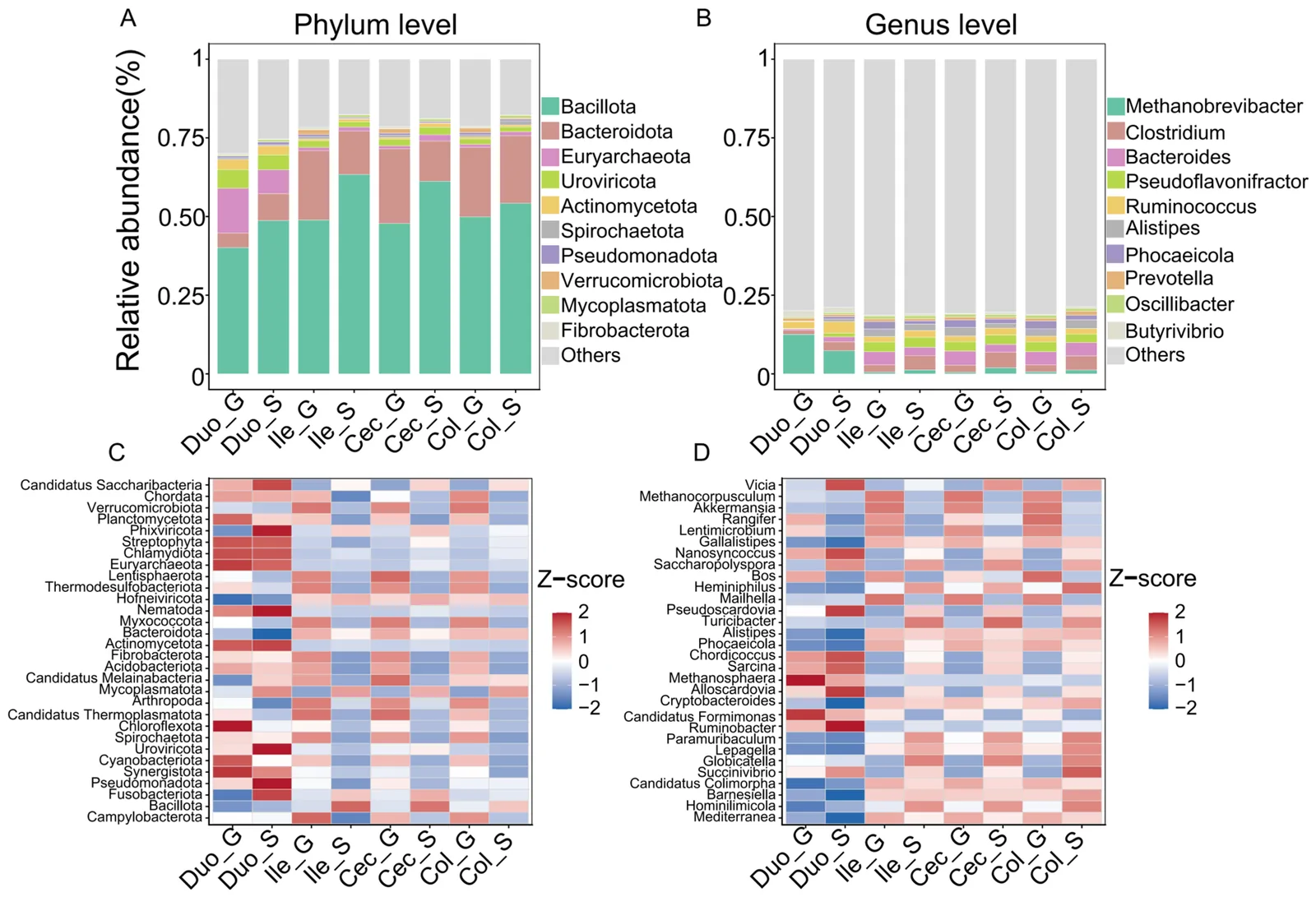
Taxonomic composition of gut microbial communities across four intestinal segments in grazing and stall-fed yaks. Shotgun metagenomic profiles were obtained from duodenal, ileal, cecal, and colonic digesta of grazing yaks (G; n = 3) and stall-fed yaks (S; n = 3). Each bar or heatmap column represents the group mean for one intestinal segment–feeding system combination. (**A**,**B**) Relative abundances (%) of the 10 most abundant phyla and genera, respectively; all remaining taxa were grouped as “Others.” Colors identify individual taxa according to the legends. (**C**,**D**) Heatmaps of the 30 phyla and genera with the greatest variance after filtering for mean relative abundance ≥0.01% and prevalence ≥25%. Colors represent row-scaled Z-scores of log10-transformed relative abundance, with red and blue indicating values above and below the mean for each taxon, respectively. The panels provide descriptive comparisons only. Duo, duodenum; Ile, ileum; Cec, cecum; Col, colon; G, grazing; S, stall-fed.

At the phylum level, group-mean relative abundance profiles showed that Bacillota and Bacteroidota were the predominant phyla across the duodenum, ileum, cecum, and colon of both grazing and stall-fed yaks (Figure 2A). Bacteroidota displayed higher group-mean relative abundances in the cecum and colon than in the proximal intestinal segments, whereas Bacillota showed variable distribution patterns along the intestinal tract. Other phyla, including Pseudomonadota, Spirochaetota, Verrucomicrobiota, and Actinomycetota, were present at lower group-mean relative abundances. Some feeding-system-associated variation in group-mean relative abundance was observed descriptively within individual intestinal segments; however, the overall phylum-level profiles were broadly similar between grazing and stall-fed yaks. At the genus level, the group-mean relative abundance profiles of the 10 most abundant genera showed greater compositional complexity than the phylum-level profiles (Figure 2B). The relative contributions of *Methanobrevibacter*, *Clostridium*, *Bacteroides*, *Pseudoflavonifractor*, *Ruminococcus*, *Alistipes*, *Phocaeicola*, *Prevotella*, *Oscillibacter*, and *Butyrivibrio* varied descriptively among intestinal segments, with additional between-group variation observed within some segments. However, because these stacked bar plots represent descriptive group means rather than inferential statistical comparisons, the observed patterns were not interpreted as statistically significant taxon-specific differences.

Heatmap analysis further illustrated segment-associated patterns in taxonomic composition (Figure 2C,D). Variance-selected phyla, including Bacteroidota, Actinomycetota, and Fibrobacterota, and the 30 genera with the greatest variance displayed heterogeneous standardized abundance patterns across the duodenum, ileum, cecum, and colon. Archaeal genera were represented mainly by *Methanocorpusculum* and *Methanobrevibacter*. These patterns were consistent with the broader segment-associated community differences identified by ANOSIM (Table 2).

### 3.3. Predicted Microbial Functional Profiles Based on CAZy and KEGG Annotations

**Figure 3:**
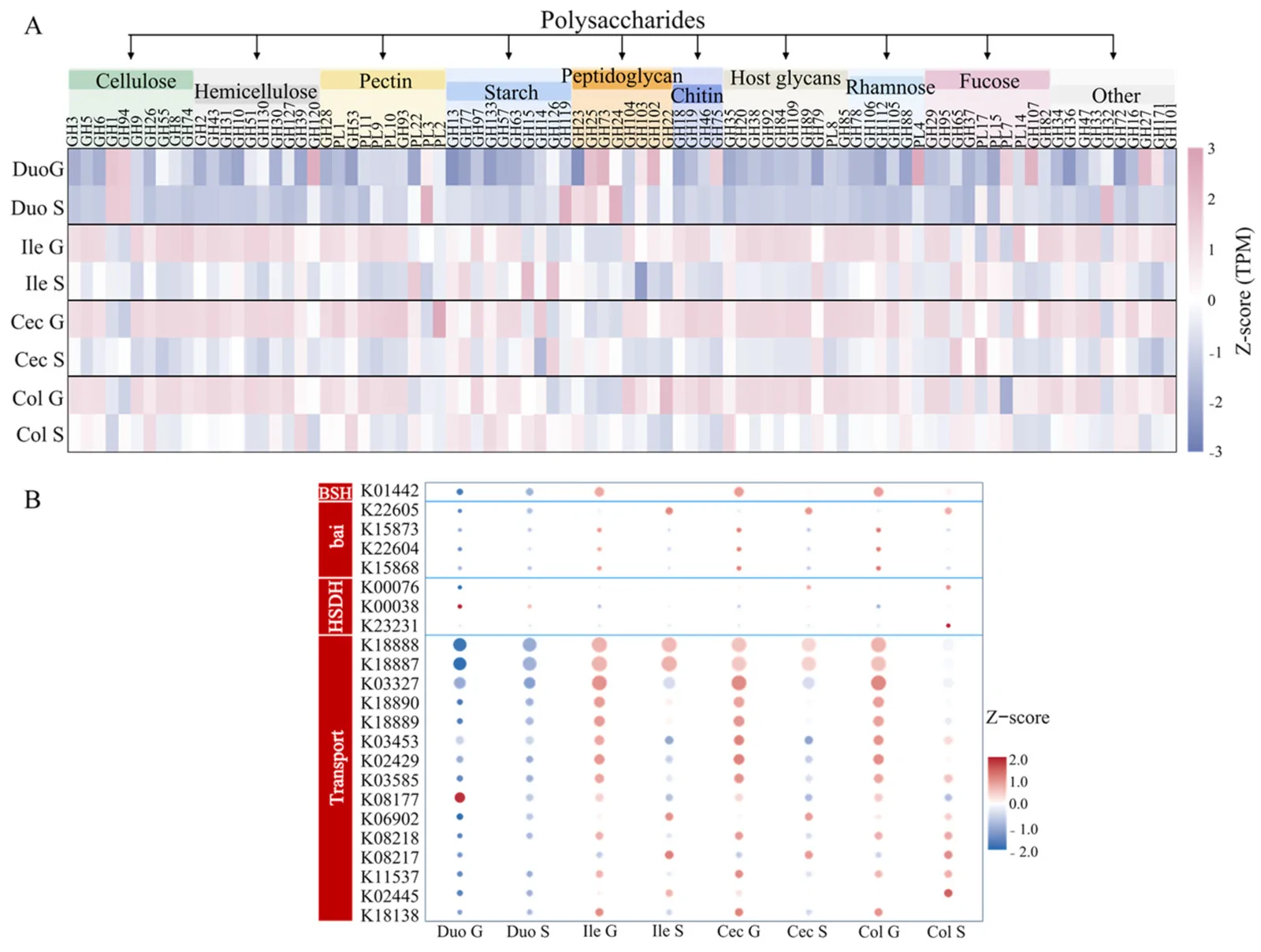
Predicted carbohydrate-active enzyme and bile-acid-related functional profiles of gut microbiota in grazing and stall-fed yaks. Shotgun metagenomic data were obtained from duodenal, ileal, cecal, and colonic digesta of grazing yaks (G; n = 3) and stall-fed yaks (S; n = 3). Each row in panel A and each column in panel B represents the group mean for one intestinal segment–feeding system combination. (**A**) Heatmap of carbohydrate-active enzyme (CAZy) families grouped by predicted substrate category. Colors indicate row-scaled Z-scores of normalized gene abundance, with red and blue representing values above and below the mean for each family, respectively. (**B**) Bubble plot of 23 bile-acid-related KEGG orthologs grouped into bile salt hydrolase, bile acid-inducible, hydroxysteroid dehydrogenase, and transport/resistance modules. Bubble size indicates mean normalized abundance, and bubble color indicates the row-scaled Z-score. Functional annotations represent predicted genomic potential rather than gene expression or enzyme activity. No confirmatory differential-abundance testing was performed for these functional profiles. Duo, duodenum; Ile, ileum; Cec, cecum; Col, colon; CAZy, carbohydrate-active enzymes; KEGG, Kyoto Encyclopedia of Genes and Genomes; KO, KEGG ortholog; BSH, bile salt hydrolase; *bai*, bile acid-inducible genes; HSDH, hydroxysteroid dehydrogenase.

The CAZy heatmap showed descriptive variation in the normalized abundances of CAZy families associated with carbohydrate metabolism across intestinal segments and feeding systems (Figure 3A). In the group-mean profiles, several CAZy families annotated as involved in structural polysaccharide utilization showed higher mean normalized abundances in the ileum, cecum, and colon of grazing yaks, whereas some starch-associated CAZy families showed higher mean abundances in stall-fed yaks, particularly in the colon. In the ileum, the mean relative abundances of GH2, GH3, and GH43 were 0.0035, 0.0029, and 0.0021, respectively, in grazing yaks, compared with 0.0026, 0.0024, and 0.0013 in stall-fed yaks. GH29 and GH101 also showed higher mean abundances in the cecum and colon of grazing yaks, whereas GH13 showed the highest group-mean abundance in the colon of stall-fed yaks. Descriptive profiling of 23 bile-acid-related KEGG orthologs, grouped into bile salt hydrolase, bile acid-inducible, hydroxysteroid dehydrogenase, and transport/resistance modules, showed predominantly higher mean normalized abundances in the cecum and colon than in the duodenum and ileum (Figure 3B). K01442 showed higher group-mean abundance in the ileum, cecum, and colon of grazing yaks, K00038 showed higher group-mean abundance in the duodenum of grazing yaks, and K23231 showed higher group-mean abundance in the colon of stall-fed yaks. Transport- and resistance-related orthologs, including K03453, K02429, K18888, and K18887, showed higher group-mean abundances in the hindgut of grazing yaks. These descriptive profiles represent predicted genomic functional potential and were not interpreted as evidence of statistically significant differential abundance, gene expression, enzyme activity, or metabolic flux.

### 3.4. Spatial Variation in Bile Acid Profiles Along the Gut–Liver Axis

**Figure 4:**
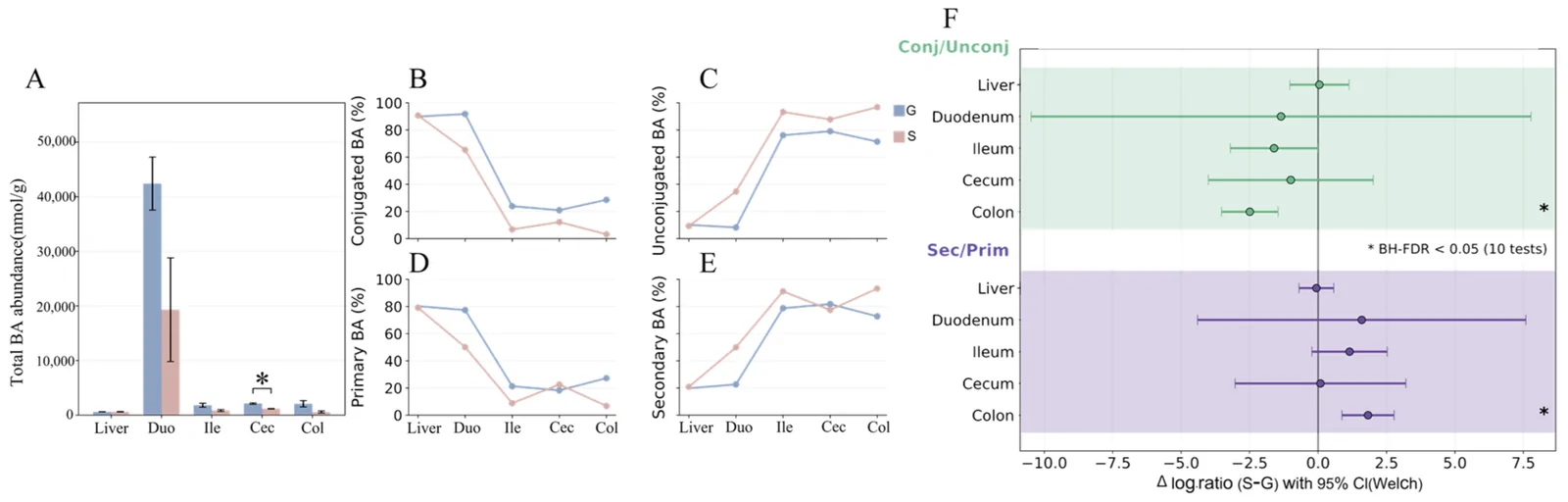
Bile acid concentrations and compositional profiles along the yak gut–liver axis in grazing and stall-fed yaks. Targeted bile acid metabolomics was performed on liver tissue and digesta from the duodenum, ileum, cecum, and colon of grazing yaks (G; n = 3) and stall-fed yaks (S; n = 3). (**A**) Total bile acid concentration (nmol/g), presented as mean ± SEM. Blue and pink bars represent grazing and stall-fed yaks, respectively. Groups were compared within each anatomical site using Welch’s *t*-tests; site-specific total bile acid comparisons were interpreted as exploratory, and * indicates an unadjusted *p* < 0.05. (**B**–**E**) Group-mean proportions of conjugated, unconjugated, primary, and secondary bile acids, respectively; blue and pink lines represent G and S. (**F**) Differences in bile acid log-ratios between feeding systems (Δlog-ratio = S-G). Points and horizontal lines represent estimated differences and 95% confidence intervals from Welch’s *t*-tests. Positive and negative values indicate higher ratios in S and G, respectively. The upper and lower panels show conjugated-to-unconjugated and secondary-to-primary bile acid ratios, respectively. *p* values from the 10 ratio comparisons were adjusted using the Benjamini–Hochberg procedure; * indicates BH-FDR < 0.05. BA, bile acid; Duo, duodenum; Ile, ileum; Cec, cecum; Col, colon; SEM, standard error of the mean; BH-FDR, Benjamini–Hochberg false discovery rate.

A total of 40 bile acids were detected in at least one liver or intestinal sample, of which 25 were consistently detected across the liver, duodenum, ileum, cecum, and colon. TαMCA and TβMCA were detected only in the liver, whereas TUDCA was detected only in the duodenum. Nine bile acids—muroCA, isoLCA, UDCA, βMCA, 7,12-DiketoLCA, βUDCA, 6-KetoLCA, αMCA, and βHDCA—were not detected in the liver. Seven bile acids—AlloCA, LCA-3S, 7,12-DiketoLCA, βUDCA, 6-KetoLCA, αMCA, and βHDCA—were not detected in the duodenum, and GUDCA was not detected in the colon (Table 4). Total bile acid concentrations were highest in the duodenum and decreased progressively toward the distal intestine in both grazing and stall-fed yaks (Figure 4A). Mean cecal total bile acid concentration was lower in stall-fed than in grazing yaks in the exploratory site-specific comparison (1169.41 ± 45.65 vs. 2125.26 ± 129.63 nmol/g, mean ± SEM; unadjusted Welch’s *p* = 0.0109), although this site-specific difference did not remain significant after correction for multiple anatomical-site comparisons. Conjugated and primary bile acids predominated in the liver and duodenum, whereas the proportions of unconjugated and secondary bile acids increased toward the hindgut (Figure 4B–E). Log-ratio analysis identified selective between-group differences in colonic bile acid composition (Figure 4F). Stall-fed yaks had a lower conjugated-to-unconjugated bile acid ratio and a higher secondary-to-primary bile acid ratio than grazing yaks, and both differences remained significant after Benjamini–Hochberg correction (BH-FDR < 0.05). Overall, bile acid composition showed a clear proximal-to-distal transition, whereas statistically supported between-group differences were limited primarily to selected colonic bile acid ratios.

### 3.5. Exploratory Associations Between Gut Microbial Genera and Bile Acids

**Figure 5:**
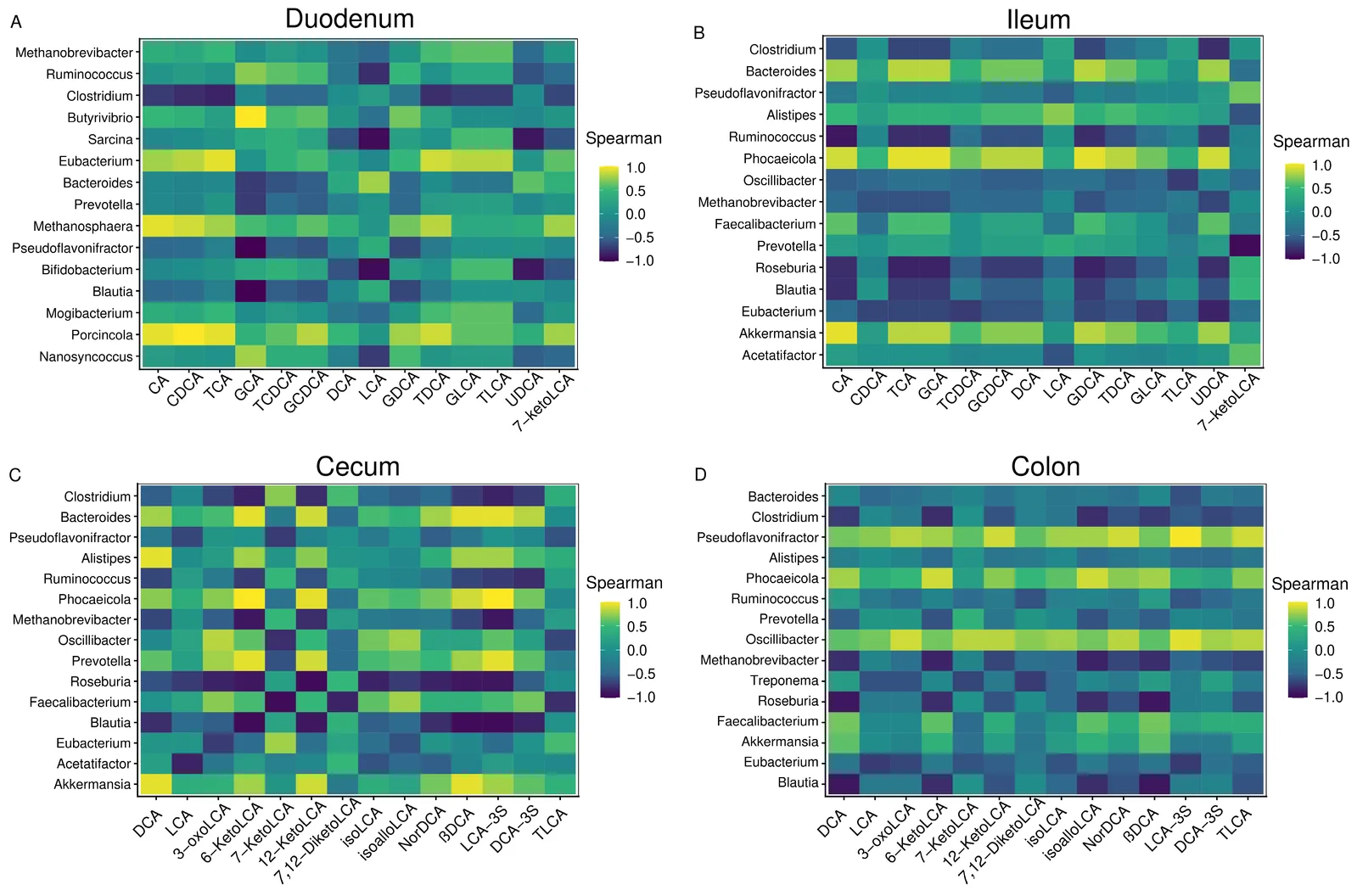
Exploratory genus–bile acid correlations across four intestinal segments in yaks. Spearman correlations were calculated separately for the duodenum (**A**), ileum (**B**), cecum (**C**), and colon (**D**) using six animals per segment, including three grazing and three stall-fed yaks. Rows show the 15 most abundant genera within each segment, and columns show bile acids selected according to segment-specific detectability and biological relevance to primary, conjugated, secondary, and related bile acid profiles. All selected bile acids were detected in at least four of the six samples, and selection was not based on statistical significance. Bile acid concentrations were transformed as log10(x + 1 × 10^−6^). Colors indicate Spearman’s ρ, with yellow–green representing positive correlations and blue–purple representing negative correlations. Because of the small sample size, the correlations are presented for descriptive and hypothesis-generating purposes only; multiple-testing-adjusted significance was not inferred, and correlation coefficients should not be interpreted as evidence of causality. CA, cholic acid; CDCA, chenodeoxycholic acid; TCA, taurocholic acid; GCA, glycocholic acid; TCDCA, taurochenodeoxycholic acid; GCDCA, glycochenodeoxycholic acid; DCA, deoxycholic acid; LCA, lithocholic acid; GDCA, glycodeoxycholic acid; TDCA, taurodeoxycholic acid; GLCA, glycolithocholic acid; TLCA, taurolithocholic acid; UDCA, ursodeoxycholic acid; 3-oxoLCA, 3-oxo-lithocholic acid; 6-KetoLCA, 6-ketolithocholic acid; 7-KetoLCA, 7-ketolithocholic acid; 12-KetoLCA, 12-ketolithocholic acid; 7,12-DiketoLCA, 7,12-diketolithocholic acid; isoLCA, isolithocholic acid; isoalloLCA, isoallolithocholic acid; NorDCA, nordeoxycholic acid; βDCA, β-deoxycholic acid; LCA-3S, lithocholic acid 3-sulfate; DCA-3S, deoxycholic acid 3-sulfate.

**Figure 6:**
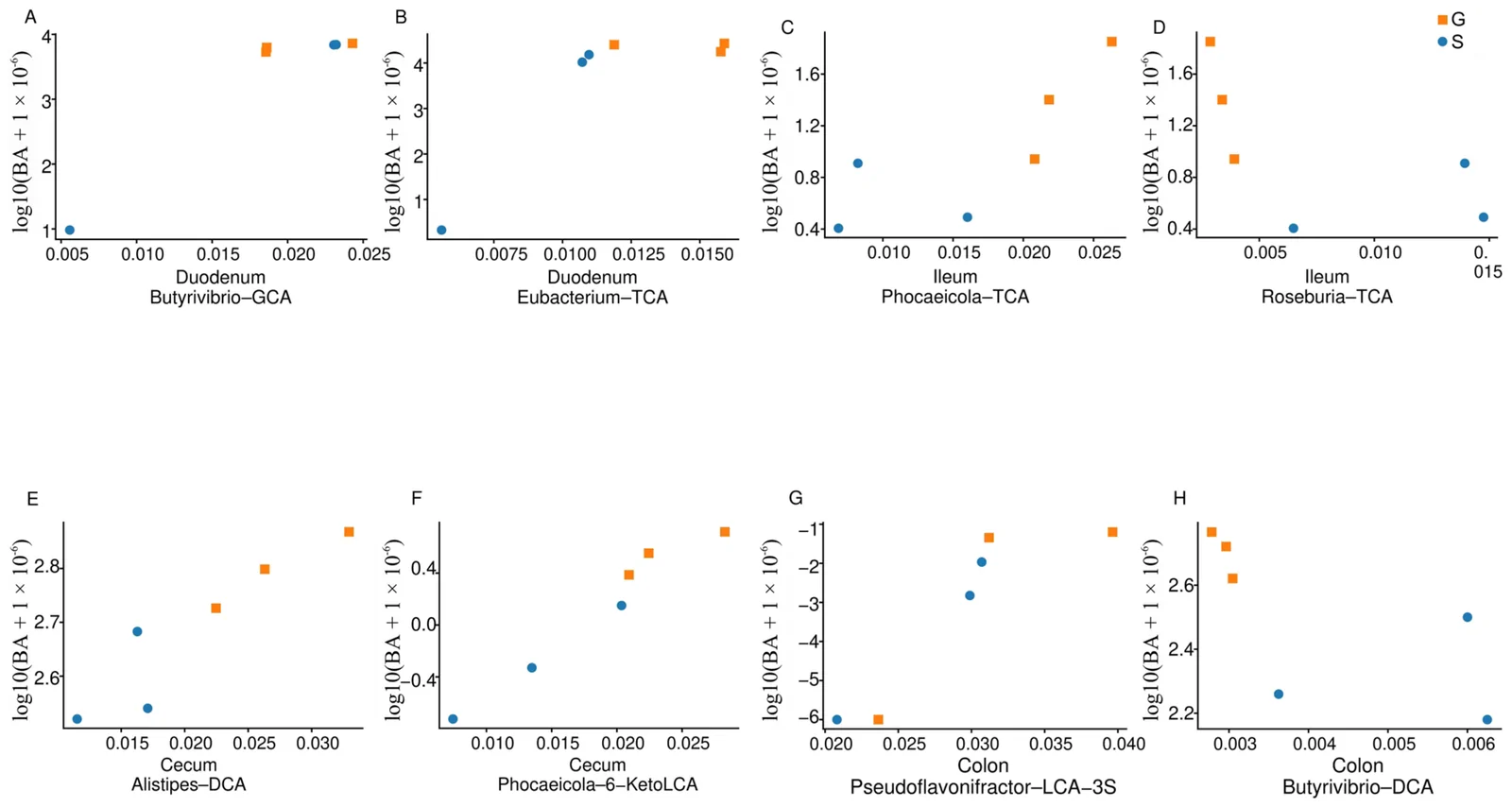
Representative exploratory genus-bile acid associations across four intestinal segments in grazing and stall-fed yaks. Each panel includes six animals from one intestinal segment, comprising three grazing yaks (G) and three stall-fed yaks (S). (**A**) Duodenal *Butyrivibrio*-GCA association; (**B**) duodenal *Eubacterium*-TCA association; (**C**) ileal *Phocaeicola*-TCA association; (**D**) ileal *Roseburia*-TCA association; (**E**) cecal *Alistipes*-DCA association; (**F**) cecal *Phocaeicola*-6-KetoLCA association; (**G**) colonic *Pseudoflavonifractor*-LCA-3S association; (**H**) colonic *Butyrivibrio*-DCA association. Orange squares indicate grazing yaks, and blue circles indicate stall-fed yaks. The x-axis shows genus relative abundance, and the y-axis shows bile acid concentration transformed as log10(x + 1 × 10^−6^). The displayed pairs were selected for descriptive illustration rather than on the basis of statistical significance. Because each segment included only six animals, these associations are exploratory and should not be interpreted as causal or statistically confirmed evidence.

Spearman’s rank correlations between the 15 most abundant genera and selected bile acids showed heterogeneous descriptive association patterns across intestinal segments (Figure 5). The duodenal and ileal profiles included correlations involving primary and conjugated bile acids, including TCA and GCA, whereas the cecal and colonic profiles included correlations involving secondary bile acids and related derivatives, such as DCA and LCA. These descriptive patterns paralleled the proximal-to-distal variation in bile acid composition but cannot distinguish microbial transformation from host synthesis, absorption, or enterohepatic circulation. Eight representative genus–bile acid pairs were further visualized using scatterplots (Figure 6). Because each segment included only six animals and multiple-testing-adjusted significance was not inferred, all correlations were treated as descriptive and hypothesis-generating. They were not interpreted as statistically robust associations or causal relationships.

## 4. Discussion

This study characterized the spatial heterogeneity of the yak gut microbiome and bile acid profiles along the gut–liver axis by integrating shotgun metagenomics and targeted metabolomics. Consistent with the recognized regional heterogeneity of mammalian intestinal ecosystems [11], our results indicate that the intestinal segment represented the major source of variation associated with microbial community composition, whereas the overall feeding-system effect was not statistically significant. PERMDISP detected no significant differences in within-group dispersion (all BH-FDR ≥ 0.1682), indicating that the observed segment-associated community separation was not accompanied by significant differences in dispersion [12]. This spatial pattern is consistent with studies showing that regional physiological environments create distinct microbial niches [13]. The proximal intestine is characterized by relatively high concentrations of primary and conjugated bile acids and rapid digesta transit, whereas the distal intestine provides a more anaerobic environment with longer retention time and greater availability of fermentable substrates [14]. Correspondingly, total bile acid concentrations were highest in the duodenum and decreased distally, while the proportions of unconjugated and secondary bile acids increased toward the hindgut [15,16]. At the microbial level, Bacteroidota showed higher mean relative abundances in the cecum and colon than in the proximal intestinal segments, whereas Bacillota displayed variable distribution patterns along the intestinal tract [17]. Because intestinal bile acid concentrations reflect the combined effects of host synthesis, secretion, absorption, enterohepatic circulation, and microbial transformation, these spatial profiles should not be interpreted as direct measures of microbial bile acid metabolic activity. Together, these findings indicate that the intestinal segment represents a major spatial context associated with microbiome–bile acid variation in the yak gut.

Within this segment-dominated framework, feeding-system-related patterns were comparatively subtle. Alpha-diversity indices did not differ significantly between grazing and stall-fed yaks within individual intestinal segments, and the overall feeding-system effect on microbial community composition was not statistically significant. This pattern is compatible with previous observations that mature gut microbial communities may maintain overall community structure despite environmental variation [18]. Nevertheless, descriptive genus-level profiles showed some between-group variation within individual intestinal segments. Because these taxonomic profiles were based on group means and were not subjected to confirmatory differential-abundance testing, they should be interpreted as exploratory patterns rather than evidence of feeding-induced microbial restructuring. The genera highlighted by abundance- and variance-based visualizations may reflect microbial configurations associated with differences in substrate availability between natural forage and stall-feeding diets [19]. The observed taxonomic patterns remained strongly segment-dependent, consistent with the significant intestinal-segment effect identified in the community-level analysis.

Descriptive CAZy profiling showed variation in the mean abundances of CAZy families annotated as related to carbohydrate utilization across intestinal segments and feeding systems. Several CAZy families annotated as related to structural polysaccharide utilization, including GH2, GH3, and GH43, showed higher group-mean abundances in grazing yaks, with these descriptive differences particularly evident in the ileum, which may be consistent with differences in dietary substrate availability between grazing and stall-feeding conditions [20]. In contrast, GH13, a CAZy family annotated as related to starch utilization, showed its highest mean abundance in the colon of stall-fed yaks [21]. This descriptive pattern is compatible with the possibility that some starch-derived substrates reached the hindgut, although starch digestion and substrate flux were not directly measured in the present study. Such a possibility is consistent with ruminant digestive physiology, in which incompletely digested starch can reach the hindgut [22]. The relatively long digesta retention time and dense microbial community of the colon may provide conditions favorable for fermentation of substrates reaching the hindgut [23]. Previous studies have also reported associations between high-starch diets and changes in colonic microbial communities and metabolite profiles in ruminants [24]. More broadly, feeding strategies that alter the balance between fibrous substrates and concentrates can influence rumen fermentation and nutrient utilization in ruminants, highlighting the broader physiological relevance of dietary substrate composition and feeding management [25]. These CAZy profiles should therefore be interpreted as descriptive indicators of predicted genomic functional potential rather than evidence of altered enzyme activity or metabolic flux.

Selective differences in colonic bile acid composition were observed between feeding systems. Stall-fed yaks had a lower conjugated-to-unconjugated bile acid ratio and a higher secondary-to-primary bile acid ratio than grazing yaks, and both differences remained significant after Benjamini–Hochberg correction (BH-FDR < 0.05). These compositional differences are consistent with a shift in the balance of bile acid forms but do not, by themselves, demonstrate enhanced microbial deconjugation or secondary bile acid transformation, because intestinal bile acid profiles reflect the combined effects of host synthesis, secretion, absorption, enterohepatic circulation, and microbial transformation [26,27]. Descriptive profiling of 23 bile-acid-related KEGG orthologs showed predominantly higher mean abundances in the cecum and colon than in the duodenum and ileum. This spatial pattern may be compatible with the ecological characteristics of the distal intestine, including greater microbial biomass and longer digesta retention time reported in ruminants [11]. However, these metagenomic profiles represent gene abundance rather than gene expression or enzyme activity. Several bile-acid-related KOs also showed descriptive between-group variation. In particular, the BSH-related ortholog K01442 showed higher mean abundance in the ileum, cecum, and colon of grazing yaks, whereas stall-fed yaks showed a lower conjugated-to-unconjugated bile acid ratio in the colon. This apparent discordance indicates that the abundance of an individual BSH-related gene was not directly coupled to bile acid composition in the present dataset. BSH enzymes may differ in substrate specificity, and their activity can also be influenced by regulatory mechanisms beyond gene abundance, including post-translational regulation [28]. Contributions from other BSH-related orthologs detected in this study, including K22605 and K15873, as well as substrate availability, local physicochemical conditions, microbial community context, and host physiological processes, may also contribute to the observed bile acid profiles, although these possibilities were not directly tested. Among the HSDH-related orthologs, K00038 showed higher mean abundance in the duodenum of grazing yaks, whereas K23231 showed higher mean abundance in the colon of stall-fed yaks. The latter occurred in the same intestinal segment in which the secondary-to-primary bile acid ratio was higher in stall-fed yaks. This co-occurrence is compatible with a possible association between predicted HSDH-related functional potential and colonic bile acid composition, but it does not demonstrate increased HSDH activity or causal involvement. Different HSDH enzymes can exhibit distinct substrate specificities and catalytic directionalities, further limiting direct inference of enzymatic activity from metagenomic gene abundance alone [29]. Transport- and resistance-related orthologs, including K03453, K02429, K18888, and K18887, also showed higher mean abundances in the hindgut of grazing yaks. These genes may represent predicted functions associated with microbial responses to the intestinal bile environment, but their higher abundance should not be interpreted as direct evidence of enhanced bile acid efflux or tolerance. Bacterial tolerance to bile involves multiple mechanisms, including membrane adaptation, stress responses, and transport-associated processes [30,31,32]. In the stall-fed colon, the higher mean abundance of the HSDH-related ortholog K23231 occurred alongside descriptive variation in the taxonomic composition of the microbial community. Previous studies in ruminants have reported associations among dietary interventions, gut microbial composition, and bile acid metabolism. Nevertheless, the present data do not allow specific taxa to be assigned as the source of the observed bile acid-related functional potential [33]. In grazing yaks, several transport- and resistance-related orthologs showed higher mean abundances in the hindgut. These descriptive patterns may reflect microbial functions involved in adaptation to the intestinal bile environment, although transporter activity and bile tolerance were not directly measured. RND-family efflux systems have been reported to participate in bacterial stress responses, including responses to bile-associated environmental pressures [34]. Experimental work in *Bacteroides thetaiotaomicron* has also shown that an RND-type pump can participate in bile-induced responses, illustrating one possible mechanism by which gut bacteria respond to the bile environment [35]. Together, these descriptive patterns suggest possible associations among feeding system, microbial taxonomic composition, predicted bile-acid-related functional potential, and bile acid profiles. However, the present data do not establish causal relationships among these features.

An important limitation of this exploratory study is the limited animal-level replication, with only three yaks included in each feeding system. The individual yak, rather than each intestinal segment, represents the independent experimental unit, and samples collected from the four intestinal segments of the same animal constitute repeated observations. Consequently, statistical power to detect feeding-system-associated differences was limited, nonsignificant results should not be interpreted as evidence of equivalence, and the small number of animals also limited permutation resolution and increased the sensitivity of segment-specific correlations to individual observations. Detailed information on pasture botanical composition, nutrient composition, and individual forage intake was unavailable, and the two feeding systems differed in both diet and management conditions; therefore, the observed between-group patterns cannot be attributed to diet alone. In addition, rumen samples were not collected, limiting interpretation of potential upstream rumen contributions to downstream intestinal patterns. The study was conducted at a single experimental site using adult male yaks and terminal endpoint sampling, which limits generalization beyond the present experimental setting. Furthermore, taxonomic profiles based on relative-abundance data are subject to compositional constraints, while CAZy and bile-acid-related KEGG profiles represent predicted genomic functional potential rather than gene expression, enzyme activity, or metabolic flux. The genus-bile acid correlations were likewise exploratory and cannot establish causality. Larger independent cohorts with longitudinal sampling and activity-based functional measurements will be required to validate these findings.

## 5. Conclusions

Overall, this exploratory multi-omics study characterized segment-specific spatial variation in the yak gut microbiome and bile acid profiles. Intestinal segment represented the major factor associated with variation in microbial community composition, whereas the overall feeding-system effect on community structure was not statistically significant. Feeding-system-associated patterns were selective and were observed mainly as descriptive differences in predicted microbial functional profiles and selected colonic bile acid compositions. Because metagenomic abundance does not measure gene expression, enzyme activity, or metabolic flux, and intestinal bile acid pools integrate host and microbial processes, the present data do not establish microbial bile acid transformation mechanisms. Larger independent cohorts with greater animal-level replication, longitudinal sampling, and activity-based functional measurements are needed to validate these exploratory patterns.

## Figures and Tables

**Table 1:** Ingredient composition and nutrient levels of the concentrate mixture fed to stall-fed yaks on a dry matter basis.

Ingredient	Amount (%)	Nutrient Level	Amount (%)
Corn	50.00	Dry Matter	94.96
Wheat	19.00	Crude Protein	15.85
Soybean Meal	20.00	Fat	2.63
Rapeseed Meal	6.00	Neutral Detergent Fiber	44.53
Calcium Carbonate	1.00	Acid Detergent Fiber	25.18
Premix ^1,2^	4.00	Calcium	0.87
Total	100.00	Phosphorus	0.46
		Metabolizable energy (MJ/kg DM)	10.61

^1^ The premix consisted of rice bran powder, salt, lysine (70%), methionine (99%), threonine (98.5%), tryptophan, choline chloride (60%), multivitamins, trace elements, citric acid, thermostable phytase, mycotoxin adsorbent, flavoring agents, and sweeteners. ^2^ The premix provided the following nutrients per kilogram of diet: Cu 10 mg, Fe 65 mg, Mn 30 mg, Zn 25 mg, I 0.5 mg, Se 0.1 mg, Co 0.1 mg, VA 4000 IU, VD 500 IU, and VE 40 IU. Metabolizable energy was calculated, whereas all other nutrient values were determined analytically.

**Table 2:** ANOSIM analysis of gut microbial community composition across intestinal segments and feeding systems.

Comparison	ANOSIM R	Permutation *p*	BH-FDR
Intestinal segment	0.2208	0.0024	0.0144
Feeding system, all segments	0.3747	0.1000	0.1200
Duodenum, G vs. S	0.1111	0.2000	0.2000
Ileum, G vs. S	1.0000	0.1000	0.1200
Cecum, G vs. S	0.8889	0.1000	0.1200
Colon, G vs. S	1.0000	0.1000	0.1200

ANOSIM was performed using the Bray–Curtis dissimilarity matrix. G denotes grazing and S denotes stall-feeding. The intestinal-segment effect was assessed using 9999 permutations restricted within animal identity because four intestinal segments were sampled from each animal. The overall feeding-system effect was assessed by permuting feeding-system labels at the animal level; thus, the 12 segment-level observations per feeding system were not treated as 12 independent animals. Feeding-system comparisons within each intestinal segment were evaluated using exact permutations. *p* values from all six ANOSIM tests were adjusted using the Benjamini–Hochberg false discovery rate procedure, and statistical significance was defined as BH-FDR < 0.05. Positive ANOSIM R values indicate that between-group dissimilarities are greater than within-group dissimilarities, with larger values indicating stronger group separation. ANOSIM, analysis of similarities; BH-FDR, Benjamini–Hochberg false discovery rate.

**Table 3:** PERMDISP analysis of within-group dispersion in gut microbial communities between grazing and stall-fed yaks.

Scope	n(G)	n(S)	Mean Distance to Centroid (G)	Mean Distance to Centroid (S)	F	Permutation *p*	BH-FDR
All segments	12	12	0.1186	0.1011	0.4267	0.7503	0.9379
Duodenum	3	3	0.0830	0.1323	1.6429	0.9504	0.9504
Ileum	3	3	0.0314	0.0439	8.3976	0.1009	0.1682
Cecum	3	3	0.0298	0.0573	11.4852	0.1009	0.1682
Colon	3	3	0.0281	0.0506	6.3763	0.1009	0.1682

G, grazing; S, stall-feeding. Digesta samples were collected from the duodenum, ileum, cecum, and colon of three animals per feeding system. Segment-specific comparisons included three animals per feeding system. The all-segment analysis included 12 segment-level observations per feeding system, comprising four intestinal segments from each of three animals; therefore, these observations do not represent 12 independent animals. PERMDISP was performed using the same Bray–Curtis distance matrix used for principal coordinate analysis. A larger mean distance to centroid indicates greater within-group dispersion. Permutation-derived *p* values were calculated using 9999 permutations and adjusted across five comparisons—the all-segment analysis and four segment-specific analyses—using the Benjamini–Hochberg false discovery rate procedure. No comparison met the significance criterion of BH-FDR < 0.05. PERMDISP evaluates differences in within-group dispersion and does not independently test differences in microbial community centroids. BH-FDR, Benjamini–Hochberg false discovery rate; PERMDISP, permutational analysis of multivariate dispersions.

**Table 4:** Targeted bile acid analytes and abbreviations used in this study.

Bile Acid Species	Name of Bile Acids	Abbreviation
CA	Glycocholic acid	GCA
	Cholic acid	CA
	Taurocholic acid	TCA
	Ursocholic acid	UCA
	Norcholic acid	NorCA
	β-Cholic Acid	βCA
	murocholic acid	muroCA
	Allocholic Acid	AlloCA
DCA	Taurodeoxycholic acid	TDCA
	Nordeoxycholic acid	NorDCA
	β-deoxycholic Acid	βDCA
	Deoxycholic acid	DCA
	Glycodeoxycholic acid	GDCA
	deoxycholic acid 3 sulfate	DCA-3S
CDCA	Taurochenodeoxycholic acid	TCDCA
	Chenodeoxycholic acid	CDCA
	Glycochenodeoxycholic acid	GCDCA
UDCA	Tauroursodeoxycholic acid	TUDCA
	Glycoursodeoxycholic acid	GUDCA
	β-Ursodeoxycholic Acid	βUDCA
	Ursodeoxycholic acid	UDCA
LCA	3-oxo-lithocholic acid	3-oxoLCA
	Isoallolithocholic acid	isoalloLCA
	Lithocholic acid	LCA
	Isolithocholic acid	isoLCA
	7-ketolithocholic acid	7-KetoLCA
	12-ketolithocholic acid	12-KetoLCA
	Glycolithocholic acid	GLCA
	Taurolithocholic acid	TLCA
	7,12-diketolithocholic acid	7,12-DiketoLCA
	6-ketolithocholic acid	6-KetoLCA
	lithocholic acid 3 sulfate	LCA-3S
MCA	Tauro α-muricholic acid	TαMCA
	Tauro β-muricholic acid	TβMCA
	β-Muricholic acid	βMCA
	α-Muricholic acid	αMCA
HCA	7-Dehydrocholic acid	7-DHCA
	12-Dehydrocholic acid	12-DHCA
	3-Dehydrocholic acid	3-DHCA
	β-hyodeoxycholic acid	βHDCA

Bile acid analytes are grouped according to their parent bile acid classes. The first column indicates the parent bile acid class, the second column provides the full analyte name, and the third column provides the abbreviation used throughout the text, tables, and figures. The table lists the analytes included in the targeted bile acid assay and does not indicate that every analyte was detected in every anatomical site. UPLC–MS/MS, ultra-performance liquid chromatography-tandem mass spectrometry.

## Data Availability

The raw metagenomic sequencing data generated in this study have been deposited in the NCBI Sequence Read Archive (SRA) under BioProject accession number PRJNA1447602. The bile acid metabolomics data are available from the corresponding author upon reasonable request.
